# Self‐collected Pap smears may provide an acceptable and effective method of cervical cancer screening

**DOI:** 10.1002/hsr2.33

**Published:** 2018-04-06

**Authors:** Amita A. Singla, Paul Komesaroff

**Affiliations:** ^1^ Department of Gynaecology The Queen Elizabeth Hospital Adelaide Australia; ^2^ Department of Obstetrics and Gynaecology University of Adelaide Adelaide Australia; ^3^ Monash University Melbourne Australia

**Keywords:** cervical cancer, colposcopy, Pap smear, screening, self‐collected test

## Abstract

**Background and aims:**

The role of the Papanicolou (Pap) smear in the early detection and prevention of cervical cancer is well established. However, many women fail to undertake the test because of embarrassment or other reasons. To address this problem, we evaluated the feasibility of implementing self‐sampling of cervical cytology as an alternative to clinician‐collected Pap smears and compared it with the gold standard of colposcopy in terms of specificity.

**Materials and methods:**

A prospective preliminary study of 40 women recruited from the colposcopy clinic of a tertiary referral hospital was undertaken. Participants were instructed in the technique of self‐sampling and asked to collect their own Pap smears. Colposcopic examinations were performed and biopsies taken, if indicated. Clinician‐collected Pap smears were performed 4 weeks later. Pairwise agreement was calculated between the outcomes of self‐collected, colposcopic, and clinician‐collected samples using the weighted κ statistic.

**Results:**

Self‐collected Pap smear had a high level of acceptability among the women, all of whom were able to collect adequate tissue. The agreement of self‐collected Pap smears with colposcopic assessment was no worse than that of clinician‐collected Pap smears (Cohen's κ statistic 0.54 [95% CI, 0.27‐0.82]; cf 0.49 [0.2‐0.78], respectively). The specificity of self‐collected Pap smears was almost identical to that of clinician‐collected samples (specificity: 86% vs 81%, respectively). Direct comparison between patient and clinician collected Pap smears showed fair agreement (κ statistic 0.38 [0.07‐0.68]). There were no adverse events in either group.

**Conclusions:**

Self‐collection of Pap smears is an effective and acceptable alternative to clinician‐collected samples and may provide a strategy for improving compliance with cervical testing programs.

## 1 INTRODUCTION

Despite the established place of Papanicolou (Pap) smears in the prevention and early detection of cervical cancer, uptake of national cervical cytology programs remains a concern (the 2‐year participation rate for the National Cervical Screening Program [NCSP] in 2009‐2010 was 57.4% of women in the target age group).^1^, ^2^ Reasons given by women for failing to attend for smears conducted by practitioners include embarrassment,^3^ physical discomfort,^4^ the invasive nature of the pelvic examination, inconvenience, and, in remote communities, personal associations with the health practitioner.^5^, ^6^, ^7^ To overcome these barriers, a more acceptable method of cervical screening is needed. Studies have previously shown that in other settings, self‐sampling is associated with better participation rates in screening programs^8^, ^9^, ^10^, ^11^, ^12^ and lower levels of dissatisfaction.

Current National Health and Medical Research Council guidelines^13^, ^14^ continue to recommend cervical cytology as the primary screening tool for cervical cancers, with the role of human papillomavirus (HPV) testing predominantly limited to verification of cure after treatment of high‐grade abnormalities. Australian guidelines recently changed: HPV as primary screening has been introduced, and self‐sampling is recommended for non‐responding women. The relevance of self‐sampling for liquid‐based cytology, allowing for further tests if required, is now even higher, because there is a need of cytology triage for women who are HPV positive after self‐sampling.^11^, ^12^, ^15^ Previously, most studies on self‐sampling of cervical screening have only been done for detection of HPV.^16^, ^17^, ^18^, ^19^, ^20^, ^21^ If it were shown to be sufficiently reliable, self‐sampling for assessment of cervical cytology could provide a useful adjunct to existing screening methods.^1^, ^12^, ^22^, ^23^

We, therefore, conducted a pilot study to evaluate the use of self‐sampling of cervical cytology, in which we compared the reliability of this technique with the sampling by a clinician and compared both to the “gold standard” of sampling under direct colposcopic examination.

## 2 METHODS

The study was performed at the Colposcopic Outpatients Clinic at The Queen Elizabeth Hospital, South Australia, a tertiary public teaching hospital, between May 2009 and March 2010. The data analysis was done in 2012.

Participants were between the ages of 20 to 69, with a median age of 38.4, presenting for a colposcopic consultation, and who responded to posters placed in the waiting room. Exclusion criteria were pregnancy, known malignancy, history of cervical glandular abnormalities, previous hysterectomy, and a physical impairment sufficient to prevent the patient from conducting the procedure. Written consent was obtained from the participants. The study was approved by the Human Research Ethics Committee at The Queen Elizabeth Hospital.

The women were informed about the method of self‐collection with the help of a diagram on their initial visits. Women were guided to find cervix using the following instructions:

1. In the toilet, put 1 foot on top of the closed toilet seat and bend forward.
2. Insert the index finger of the nondominant hand in the vagina to feel cervix—which feels like the tip of a nose.
3. Once you feel the tip, insert the Cytobroom with dominant hand along the finger till you reach the cervix and rotate it twice.
4. Remove the Cytobroom, and put the tip into the ThinPrep jar provided.

Colposcopic examinations were then performed by the clinician/investigator and biopsies were taken, if indicated.

The women were then asked to attend a review appointment 4 weeks later to discuss the results and to undergo a conventional Pap smear, performed by their regular attending clinicians. At this visit, the sample was collected by introducing a cervibroom into the endocervix. The self‐collected and the physician‐collected specimens were sent for analysis to the same laboratory on the day of collection.

A pathologist, blinded to the method of collection, reviewed the cervical cytology. The results of the cervical cytology collected, and both reports, were reviewed by the investigators following the review, to ascertain the extent of agreement or disagreement between the results of the patient‐collected Pap smear with that collected by the clinician. Squamous abnormalities were classified according to the Australian modified Bethesda AMBS 2004 into the following 4 categories: “negative,” “low grade squamous intraepithelial lesion” (LSIL), “high grade squamous intraepithelial lesion” (HSIL), and “squamous carcinoma.” Any abnormality confirmed by colposcopy was managed according to NHRMC Guidelines.^13^

Pairwise agreement between the numbers of abnormalities identified by self‐collected, colposcopic, and clinician‐collected samples, and 95% confidence intervals were computed. Chance‐corrected agreement was assessed by weighted κ statistics.^24^ A κ value of above 0.8 was taken to indicate “very good agreement”; between 0.6 and 0.8, “reasonable agreement”; 0.4 and 0.6, “moderate agreement”; 0.2 and 0.4, “fair agreement”; and less than 0.2, “poor agreement.”^25^ Calculations were performed using SAS version 9.3.

## 3 RESULTS

A total of 40 women participated in the study. One woman was excluded because of non‐attendance at the follow‐up visit, resulting in a final sample size of 39. The median age was 38.4 years, with the range being 20 to 69. A relevant abnormality was reported in 16 out of 39 cases (13 LSIL; 3 HSIL) for the self‐collected Pap smears, in 10 out of 39 cases (6 LSIL; 4 HSIL) for clinician‐collected smears, and in 17 of 39 cases (13 LSIL; 4 HSIL) after colposcopy. A summary of the results is shown in Table 1, and pairwise comparisons are presented in Table 2. Importantly, no high‐grade abnormality identified from clinician‐collected or colposcopic samples was missed by the self‐collected sampling.

**Table 1:** Summary of outcomes of patient‐ and clinician‐collected smears with colposcopy impression

	Patient‐Collected Specimen	Clinician Collected Specimen	Colposcopy
Normal	23	29	22
LSIL	13	6	13
HSIL	3	4	4
Total	39	39	39

Abbreviations: HSIL, high‐grade squamous intraepithelial lesion; LSIL, low‐grade squamous intraepithelial lesion.

**Table 2:** Detailed pairwise comparison of outcomes of patient‐ and clinician‐collected smears with colposcopy impression

Patient No.	Patient‐Collected Smear	Clinician‐Collected Smear	Colposcopy
1	LSIL	N	NAD
2	N	N	NAD
3	N	N	NAD
4	PLSIL	N	NAD
5	PHSIL	N	HSIL
6	LSIL	N	NAD
7	N	N	Meno clinic not done
8	N	N	NAD
9	PLSIL	LSIL	LSIL
10	N	N	NAD
11	N	LSIL	N
12	LSIL	PHLSIL	LSIL
13	N	N	LSIL
14	N	N	NAD
15	N	N	LSIL
16	PLSIL	PLSIL	LSIL
17	LSIL	LSIL	LSIL
18	LSIL	N	HPV
19	N	N	Self was first ever pap not done
20	PHSIL	N	HSIL
21	N	N	N
22	N	N	Overdue for pap not done
23	N	HSIL	LSIL
24	N	LSIL	HSIL
25	PLSIL	N	NAD
26	N	N	Not done
27	PLSIL	N	NAD
28	N	N	NAD
29	N	HSIL	NAD
30	N	N	NAD
31	N	N	Not done
32	LSIL	N	LSIL
33	N	N	NAD
34	LSIL	PLSIL	LSIL
35	N	N	
36	N	HSIL	LSIL
37	PHSIL	N	HSIL
38	N	N	LSIL
39	LSIL	N	LSIL

Abbreviations: HPV, human papillomavirus; HSIL, high‐grade squamous intraepithelial lesion; LSIL, low‐grade squamous intraepithelial lesion.

The overall agreement between detection of any abnormality of the self‐collected Pap smear with that collected by a clinician was “fair” κ = 0.38 (95% CI, 0.07‐0.68). The number of patients rated as negative for any cytological abnormality was 25 after self‐sampling and 26 following clinician‐collected Pap smears.

With colposcopy taken as the gold standard, specificity, positive predicted value, and negative predictive values of both clinician‐ and self‐collected Pap smears were calculated. Comparison of clinician‐collected Pap smears with colposcopy showed a κ statistic of 0.49, indicating “moderate” agreement (CI, 0.20‐0.78), with specificity being 81%. The comparison of self‐collected paper smears with the same gold standard, on the other hand, showed a κ statistic of 0.54, indicating “moderate” agreement (CI, 0.27‐0.82). Specificity of the patient‐collected Pap smear was 86.4%.

In the past, cervical sampling was considered adequate if endocervical cells were detected; however, a definition of “adequate sample” remains elusive. More recent research has shown that the absence of endocervical cells is not necessarily associated with a higher risk of cervical abnormality.^26^, ^27^, ^28^, ^29^, ^30^, ^31^, ^32^ In our study, endocervical component was detected in 15% of self‐collected Pap smears and in 94% of clinician‐collected smears, a difference that was statistically significant (*P* < .05).

## 4 DISCUSSION

The incidence of cervical cancer has declined markedly since the introduction of cervical cytology screening programs.^2^ In Australia, both the incidence and mortality of cervical cancer almost halved following the introduction of the NCSP in 1991, achieving a historic low of 9 new cases and 2 deaths per 100 000 women per year in 2002, where it has remained ever since,^33^ a result assisted by the advent of HPV vaccination. It is believed that regular Pap tests can reduce the risk of being diagnosed with cervical cancer by up to 96%, which in Australia translates to 1200 fewer deaths each year.^2^ Nine out of 10 women diagnosed with cervical cancer in Australia have not had regular Pap smears.^34^ Conventional cervical cytology screening, therefore, remains the primary method of detection of cervical cancer.^9^, ^13^

Despite the successes, significant deficiencies in the system remain. In 2009 and 2010, the participation rate^13^ in the NCSP was only 57%. The incidence of cervical cancer in Indigenous Australian and Torres Strait Islander women was more than twice that of nonindigenous women, and mortality of Indigenous and Torres Straight Islander women was 5 times the rate for nonindigenous women.^2^ While geographic location was not a significant variable, participation rates varied markedly across socioeconomic groups, ranging from 52% among women from low socioeconomic to 63% in higher socioeconomic areas.^2^, ^13^

The proven effectiveness of the cervical screening smear program, combined with the nonparticipation rate of around 40%, suggests the need for novel methods to increase screening coverage.^7^ This study suggests that self‐sampling might have a role to play in this process.^3^ All the participants in this study found the technique of self‐sampling acceptable, comfortable, and easy to learn. Many women may prefer a test that can be performed by the woman herself, if necessary in the privacy of her own home.^3^, ^4^, ^5^, ^6^, ^9^, ^22^ Self‐sampling may also obviate known obstacles to participation in screening, such as embarrassment or discomfort within the clinical relationships.

For our study, we used cervibroom, a standard brush used in gynecology clinics. There have been few other devices like solopap, dacronswabs, and digene conical collection used for this test as well.^19^, ^20^, ^35^, ^36^ The cervibroom is already used for pap smears and comes at no extra cost; it is also easy to use. The most important difference is that with cervibroom‐collected samples, a liquid‐based cytology slide can be made if required. All of these facts make it potentially a better device to use. It is also true that the difference in HPV prevalence between the self‐collected and clinician‐collected sampling was not significantly different from 0, regardless of the sampling devices and diagnostic methods used.^19^

Our findings show that the agreement of the results of self‐sampling with the gold standard of colposcopically guided sampling is no worse than that of physician sampling. In addition, the specificity for the detection of abnormalities of self‐sampling and clinician sampling are very similar. Accordingly—at least in the cases of women otherwise disinclined to undergo sampling at all—self‐sampling would seem to be an acceptable and effective alternative that might be recommended. This technique might offer an effective alternative approach for at least some of the women who do not presently comply with the NCSP.^21^, ^22^, ^23^

The single significant difference between the 2 techniques—the lower presence of endocervical cells in the self‐collected samples—is a potential cause for concern, because it suggests that many of these samples might not have included cells from the transformation zones.^2^ This is to be expected, simply because of the more limited precision associated with self‐insertion of the cervibrush. However, there is uncertainty about the importance of obtaining endocervical cells to ensure an adequate sample for diagnostic purposes.^26^ Indeed, at least 1 study^37^ has shown no statistical significance in detection of high‐grade abnormalities in patient samples containing an endocervical in comparison with those that do not. More recent research^26^, ^27^, ^28^, ^29^, ^30^, ^31^, ^32^, ^38^, ^39^ has shown that the absence of endocervical cells is not necessarily associated with a higher risk of cervical abnormality.

This study has several limitations: It is small in size, the participants were women from a single urban center rather than from a variety of social and geographical backgrounds, and all had presented because of a prior commitment to undergoing Pap smears. Further, per our study design, the clinically collected specimen was collected 4 weeks after both the self‐test and the colposcopy (and biopsy). As sensitivity of a test performed after biopsy can be lower because of, for instance, healing, we could not compare sensitivity in our study. Future studies, with larger sample sizes, should address this in the future. In any case, the Pap smear per se has low sensitivity.^19^ Lastly, we did not directly test the hypothesis that women who otherwise fail to participate in screening programs might find self‐sampling an acceptable alternative.

In spite of these limitations, the favorable results of this study are promising and support further work to assess the place of cervical self‐sampling in the future prevention of cervical cancer in Australia. It is possible that this technique will prove a useful addition to existing strategies and contribute to increasing the participation rate in cervical screening and, therefore, to the further reduction of rates of cervical cancer.

## CONFLICTS OF INTEREST

None declared.

## AUTHOR CONTRIBUTIONS

Conceptualization: Dr Amita A. Singla

Formal analysis: Dr Amita A. Singla

Investigation: Dr Amita A. Singla

Methodology: Dr Amita A. Singla

Project administration: Dr Amita A. Singla

Writing – original draft preparation: Dr Amita A. Singla, Dr Paul Komesaroff

Writing – review and editing: Dr Amita A. Singla, Dr Paul Komesaroff

## References

[1] Budge M , Halford J , Haran M , Mein J , Wright G . Comparison of a self‐administered tampon thinprep test with conventional pap smears for cervical cytology. Aust NZ J Obstetrics Gynaecol. 2005;45(3):215‐219. 10.1111/j.1479-828X.2005.00392.x 15904447

[2] [No author] . Prevention of cervical cancer. *Cancer Council Aust*: https://www.ncbi.nlm.nih.gov/pmc/articles/PMC2736844/ [Accessed March 2012]

[3] Jones H , Wiegerinck M , Nieboer T , Mol B , Westhoff C , et al. Women in the Netherlands prefer self‐sampling with a novel lavaging device to clinician collection of specimens for cervical cancer screening. Sex Transm Dis. 2008;35(11):916‐917. 18665020 10.1097/OLQ.0b013e3181812cf0

[4] Barbee L , Kobetz E , Blanco J . Discovering a culturally appropriate alternative for Haitian American Women: the Fournier self‐sampling device. American Public Health Association abstract: http://www.health.gov.au/internet/screening/publishing.nsf/Content/cervical-screening-1 (Accessed 15th January 2015).

[5] Dzuba IG , Diaz EY , Allen B , et al. The acceptability of self‐collected samples for HPV testing vs. the Pap test as alternatives in cervical cancer screening. J Womens Health Gend Based Med. 2002;11(3):265‐275. 11988136 10.1089/152460902753668466

[6] Fylan F . Screening for cervical cancer: a review of women's attitudes, knowledge, and behaviour. Br J Gen Pract. 1998;48(433):1509‐1514. 10024713 PMC1313202

[7] Girgis A , Bonevski B , Perkins J , Sanson‐Fisher R . Self‐reported cervical screening practices and beliefs of women from urban, rural and remote regions. Obstet Gynaecol. 1999;19(2):172‐179. 10.1080/01443619965543 15512264

[8] Sanner K , Wikstrom I , Strand A , Lindell M , Wilander E . Self‐sampling of the vaginal fluid at home combined with high‐risk HPV testing. Brit J Cancer. 2009;101:871‐874. 19654577 10.1038/sj.bjc.6605194 PMC2736844

[9] Gok M , Heideman DA , van Kemenade FJ , et al. HPV testing on self‐collected cervicovaginal lavage specimens as screening method for women who do not attend cervical screening: cohort study. BMJ. 2010; Mar 11;340(mar11 1):c1040. 20223872 10.1136/bmj.c1040 PMC2837143

[10] Molnar P , Biringer A , McGeer A , McIsaac W . Can pregnant women obtain their own specimens for group B streptococcus? A comparison of maternal versus physician screening. The Mount Sinai GBS screening group. Fam Pract. 1997;14(5):403‐406. 9472376 10.1093/fampra/14.5.403

[11] Karjalainen et al. Self‐sampling in cervical cancer screening: comparison of a brush‐based and a lavage‐based cervicovaginal self‐sampling device. BMC Cancer. 2016;16(1):221 10.1186/s12885-016-2246-9 26979237 PMC4791879

[12] Mullins R , et al. Self‐sampling for cervical screening: could it overcome some of the barriers to the Pap test? J Med Screen. 2014;21(4):201‐2016. 10.1177/0969141314555247 25312640

[13] AIHW 2011 Cervical screening in Australia 2008‐2009. Cancer series no. 61. Cat. no. CAN 57. Canberra: AIHW.. Available from: http://www.aihw.gov.au/publication-detail/?id=10737420251 [accessed 12^th^ January 2015].

[14] Warren JB , Gullett H , King VJ . Cervical cancer screening and updated Pap guidelines. Primary Care: Clinics in Office Practice. March 2009;36(1):131‐149. 19231606 10.1016/j.pop.2008.10.008

[15] Tomljenovic L , Shaw CA . Human papillomavirus (HPV) vaccine policy and evidence‐based medicine: are they at odds? Ann Med. 2013;45(2):182‐193. 22188159 10.3109/07853890.2011.645353

[16] Ronco G , Giorgi‐Rossi P , Carozzi F , et al. Efficacy of human papillomavirus testing for the detection of invasive cervical cancers and cervical intraepithelial neoplasia: a randomised controlled trial. Lancet. 2010;11(3):249‐257. 20089449 10.1016/S1470-2045(09)70360-2

[17] Petignat P , Faltin DL , Bruchim I , Tramer MR , Franco EL , Coutlee F . Are self‐collected samples comparable to physician‐collected cervical specimen for human papillomavirus DNA testing? A systematic review and meta‐analysis. Gynecol Oncol. 2007;105(2):530‐535. 17335880 10.1016/j.ygyno.2007.01.023

[18] Schmeink CE , Bekkers RL , Massuger LF , Melchers WJ . The potential role of self‐sampling for high‐risk human papillomavirus detection in cervical cancer screening. Rev Med Virology. 2011;21(3):139‐153. 21538664 10.1002/rmv.686

[19] Sultana F , English DR , Simpson JA , et al. Home‐based HPV self‐sampling improves participation by never‐screened and under‐screened women: results from a large randomized trial (iPap) in Australia. Int J Cancer. 2016;139(2):281‐290. 26850941 10.1002/ijc.30031

[20] Gupta S , et al. Self‐Sampling for HPV testing: increased cervical cancer screening participation and incorporation in international screening programs. https://www.preprints.org/ > https://www.preprints.org/subject/browse/life_sciences > https://www.preprints.org/subject/browse/life_sciences/microbiology > doi: 10.20944/preprints201711.0199.v1 Preprint Article *Version 1* PMC5900042 29686981

[21] Gyllensten U , Sanner K , Gustavsson I , Lindell M , Wikström I , Wilander E . Short‐time repeat high‐risk HPV testing by self‐sampling for screening of cervical cancer. Br J Cancer. 2011;105(5):694‐697. 21811250 10.1038/bjc.2011.277 PMC3188941

[22] Elwood Martin R . Is it feasible for women to perform their own Pap smears? A research question in progress: commentary. CMAJ. 2000;162(5):666‐667. 10738455 PMC1231224

[23] Clinician‐collected versus patient‐collected cervical Pap smears. [internet] Government, Health Authority: United States: Federal :https://clinicaltrials.gov/ct2/show/NCT01214330 [accessed 12th Janaury 2015].

[24] Cohen J . Weighted kappa: nominal scale agreement with provision for scaled disagreement or partial credit. Psychol Bull. 1968;70(4):213‐220. 19673146 10.1037/h0026256

[25] Elwood M . Critical appraisal of epidemiologic studies and clinical trials. New York: Oxford University Press; 2007.

[26] Mitchell H , Medley G . Longitudinal study of women with negative cervical smears according to endocervical status. Lancet. 1991;337(8736):265‐267. 1671112 10.1016/0140-6736(91)90870-u

[27] Mitchell H , Medley G . Influence of endocervical status on the cytologic prediction of cervical intraepithelial neoplasia. Acta Cytol. 1992;36(6):875‐880. 1449025

[28] Mitchell H , Medley G . Cytological reporting of cervical abnormalities according to endocervical status. Br J Cancer. 1993;67(3):585‐588. 8439508 10.1038/bjc.1993.107 PMC1968260

[29] Sidawy MK , Tabbara SO , Silverberg SG . Should we report cervical smears lacking endocervical component as unsatisfactory? Diagn Cytopathol. 1992;8(6):567‐570. 1468332 10.1002/dc.2840080605

[30] Bos AB , van Ballegooijen M , Elske van den Akker‐van Marle M , et al. Endocervical status is not predictive of the incidence of cervical cancer in the years after negative smears. Am J Clin Pathol. 2001;115(6):851‐855. 11392881 10.1309/RP84-MD34-8MFN-39UR

[31] Mitchell HS . Longitudinal analysis of histologic high‐grade disease after negative cervical cytology according to endocervical status. Cancer. 2001;93(4):237‐240. 11507695 10.1002/cncr.9035

[32] Selvaggi SM , Guidos BJ . Endocervical component: is it a determinant of specimen adequacy? Diagn Cytopathol. 2002;26(1):53‐55. 11782089 10.1002/dc.10019

[33] National Cervical Screening Program [Internet]. Australian Government: Department of Health : https://www.ncbi.nlm.nih.gov/pubmed/21538664 [accessed 12th January 2015].

[34] Andrae B , Kemetli L , Sparén P , et al. Törnberg screening‐preventable cervical cancer risks: evidence from a nationwide audit in Sweden. J Natl Cancer Inst. 2008;100(9):622‐629. 18445828 10.1093/jnci/djn099

[35] O'Callaghan M . Military hospital clinician‐collected versus patient‐collected cervical Pap smears (SoloPaP) http://clinicaltrials.gov identifier: NCT01214330 first posted: October 5, 2010 Last Update Posted: November 21, 2014

[36] Peyton CL , Schiffman M , Lörincz AT , et al. Comparison of PCR‐ and hybrid capture‐based human papillomavirus detection systems using multiple cervical specimen collection strategies. J Clin Microbiol. 1998 Nov;36(11):3248‐3254. 9774574 10.1128/jcm.36.11.3248-3254.1998 PMC105310

[37] Huang A , Quinn M , Tan J . Outcome in women with no endocervical component on cervical cytology after treatment for high‐grade cervical dysplasia. Aust NZ J Obstetrics Gynaecol. 2009 Aug;49(4):426‐428. 10.1111/j.1479-828X.2009.01014.x 19694701

[38] Farhana S et al. High‐grade cervical abnormalities and cervical cancer in women following a negative Pap smear with and without an endocervical component: A cohort study with 10 years of follow‐up. IJC. 01 September 2014;135(5):1213‐1219. 10.1002/ijc.28756 24488882

[39] McCredie MR , Sharples KJ , Paul C , et al. Natural history of cervical neoplasia and risk of invasive cancer in women with cervical intraepithelial neoplasia 3: a retrospective cohort study. Lancet Oncol. 2008;9(5):425‐434. 18407790 10.1016/S1470-2045(08)70103-7

